# Intraoperative Endoscopic-Guided Bowel Resection for Persistent Gastrointestinal Bleeding Caused by Angiodysplasia: A Case Report and Literature Review

**DOI:** 10.1055/s-0043-1776111

**Published:** 2023-10-19

**Authors:** Emily Fellows, Joy Harris, Tania Kibble, Nicholas M. McDonald, Nabeel Azeem, James V. Harmon

**Affiliations:** 1 Department of Surgery, University of Minnesota, Minneapolis, Minnesota; 2 Division of Gastroenterology, Department of Internal Medicine, University of Minnesota, Minneapolis, Minnesota

**Keywords:** angiodysplasia, intraoperative endoscopy, bowel resection, gastrointestinal hemorrhage

## Abstract

Gastrointestinal angiodysplasia is an uncommon condition often associated with significant gastrointestinal bleeding that is resistant to medical therapy. We report the clinical outcomes of two patients who successfully underwent simultaneous intraoperative endoscopic and surgical interventions for the treatment of angiodysplasia. Intraoperative endoscopic guidance was found to be useful in managing hemorrhage caused by angiodysplasia in both patients. Additionally, we performed an analysis of cases reported in the literature. Our review focused on the anatomic location of the resected bowel and the clinical outcomes of patients ( *n* = 21) with angiodysplasia managed with intraoperative endoscopy reported in the literature.

## Introduction

Gastrointestinal angiodysplasia (GIAD) is an uncommon condition characterized by acquired vascular malformations that vary in size and clinical presentation, ranging from occult bleeding to significant gastrointestinal hemorrhage. In 90% of patients, first-occurrence angiodysplasia resolves spontaneously; however, patients with multiple comorbidities commonly require ongoing medical therapy to prevent rebleeding. 1 The usual medical treatment strategies include the administration of somatostatin analogs and antiangiogenics. In addition, some patients may require endoscopic interventions such as argon plasma coagulation (APC), mechanical clip placement, multipolar electrocoagulation, and laser photoablation. Interventional radiology with embolization may be required in some patients. 1 If endoscopic and interventional procedures fail to control hemorrhage, endoscopic-guided surgical resection can be considered. 2 3 4 5 6 Although this approach has been previously described, there are few reports of recalcitrant gastrointestinal bleeding associated with angiodysplasia requiring intraoperative endoscopic guidance for bowel resection.

We report two patients with recurrent bleeding associated with GIAD who were refractory to standard treatments, and who eventually required exploratory laparotomy, intraoperative endoscopic guidance, and bowel resections.

## Methods

We performed a retrospective case review of two patients with gastrointestinal hemorrhage who required endoscopic-guided bowel resection. Both patients provided written consent for publication of these case reports based on the Surgical CAse REport (SCARE) criteria. 7 Additionally, we performed an analysis of similar cases reported in the literature. The following keywords were used: “angiodysplasia,” “bowel resection,” and “endoscopy.” In total, 47 studies from 2000 to 2020 were discovered. Among them, five case reports and case series described the use of intraoperative-guided bowel resection for treatment of recalcitrant angiodysplasia. Demographic data, clinical comorbidities, and rebleeding rates were analyzed ( Table 1 ).

**Table 1:** Summary of previous studies that have reported the utilization and success of bowel resection with intraoperative endoscopy for bleeding angiodysplastic lesions since 2000

Study	No. of patients	Age (y)	Sex	History of aortic stenosis	Location of angiodysplasia	Procedure(s) performed (OR/LR/SILS)	Length resected (cm)	Outcomes including complications
Tartaglia et al 2	4	91	M	No	50 cm from the ligament of Treitz	OR with intraoperative enteroscopy	10	No rebleeding at 2 mo
		88	M	No	30 and 120 cm from the ligament of Treitz		100	No rebleeding at 9 mo
		83	M	No	15–220 cm from the ligament of Treitz		206	Rebleeding at 8 mo requiring transfusions
		75	F	No	80–120 cm from the ligament of Treitz		90	No rebleeding at 1 mo
Lee et al 3	1	60	M	No	Jejunum	LR with intraoperative enteroscopy	120	No rebleeding at several months
Martinez et al 4	1	82	F	No	Jejunum	LR with intraoperative enteroscopy	20	No rebleeding at 20 mo
Uhm et al 5	1	42	F	No	Jejunum, 70 cm from the ligament of Treitz	Bowel resection with intraoperative enteroscopy	100	No rebleeding at 12 mo
Douard et al 6	14	N/A	N/A	N/A	Small intestine: 11 Colon: 3	Bowel resection with intraoperative enteroscopy	N/A	1 of 11 patients who underwent small bowel resection had rebleeding postoperatively; none of the 3 patients with colon resection had rebleeding

Abbreviations: LR, laparoscopic resection; OR, open resection; N/A, not applicable; SILS, single incision laparoscopic surgery.

## Case 1

**Fig. 1:**
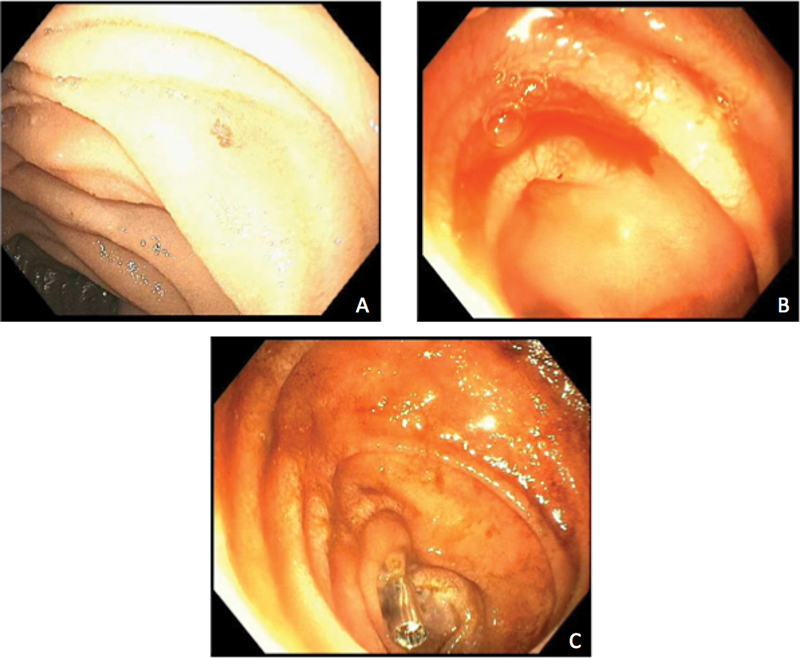
Photographs taken during intraoperative enteroscopy (case 1). ( **A** ) Multiple angiodysplastic lesions on intraoperative endoscopy. ( **B** ) Active bleeding from an angiodysplastic lesion was observed. ( **C** ) Bleeding resolved after combined argon plasma coagulation (APC) treatment and mechanical clipping. A tattoo was applied to identify the lesion for future reference.

A 68-year-old man with end-stage renal disease requiring intermittent hemodialysis presented with recurrent gastrointestinal bleeding from proximal small bowel angiodysplasia. Outpatient medical management, including twice-weekly blood transfusions, intravenous iron, and administration of erythropoietin, was not successful against recurrent bleeding. Due to an unsuccessful medical management, the patient eventually required endoscopic therapy for recurrent proximal small bowel angiodysplasia. The endoscopic interventions included through-the-scope HemoClip (Boston Scientific, Marlborough, MA) placement and ablation with APC. These interventions failed, necessitating therapeutic placement of an over-the-scope clip (OTSC) (Ovesco Endoscopy, Cary, NC). The patient's comorbidities included chronic systolic congestive heart failure, type II diabetes mellitus, alcohol use disorder, Barrett's esophagus, Crohn's disease, and chronic anemia. At presentation, the patient required a blood transfusion in the setting of a hemoglobin level of 6.9 g/dL; during the next several weeks, the patient continued to require daily blood transfusions and administration of aminocaproic acid. As the patient had multiple diffuse angiodysplastic lesions without a clear single bleeding source, interventional radiologic embolization was not pursued. Due to ongoing hemorrhage, the patient underwent multiple endoscopic interventions including the application of an OTSC for jejunal angiodysplasia ( Fig. 1 ). After OTSC placement, the patient developed left-sided abdominal pain and peritoneal signs. An abdominal computed tomography (CT) scan demonstrated intraperitoneal free fluid and free air consistent with microperforation of the jejunum at the OTSC site. The patient underwent an emergent exploratory laparotomy with intraoperative endoscopy. A segmental resection of 40 cm of jejunum was performed. A 15-mm laparoscopic port was positioned into the open lumen of the distal small bowel and secured with 0 silk sutures to create an airtight seal. The sterile endoscope was then passed through the 15-mm laparoscopic port into the bowel to permit carbon dioxide gas to distend the bowel and allow careful examination of the distal limb of the small bowel. Intraoperative endoscopic evaluation revealed that most-all angiodysplasia lesions were resected. However, several small, nonbleeding arteriovenous malformations (AVMs) were identified in the distal small bowel. A primary anastomosis was completed at 15 cm from the ligament of Treitz. Following endoscopy, the bowel remained significantly distended, prohibiting fascial closure and requiring the placement of a temporary abdominal dressing. The patient returned to the operating room on postoperative day 2 for a washout of the abdomen and closure of the abdomen. His postoperative course was complicated by the development of a small abdominal fluid collection, which resolved after percutaneous drain placement. The patient was discharged from the hospital after 30 days. No further gastrointestinal bleeding was observed at the 1-year follow-up.

## Case 2

**Fig. 2:**
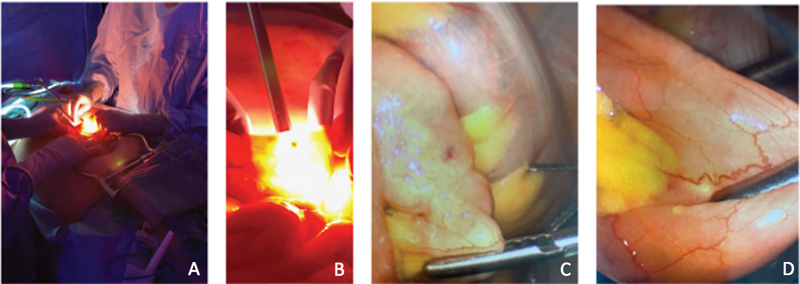
Intraoperative images of the surgical team performing bowel resection (case 2). ( **A, B** ) Surgical team performing transillumination-guided open bowel resection. ( **C, D** ) Visualization of angiodysplastic lesions during bowel resection.

An 82-year-old woman with a history of aortic stenosis and ischemic stroke on clopidogrel presented with weakness and severe anemia. Her comorbidities included coronary artery disease, chronic diastolic heart failure, stage III chronic kidney disease, and type II diabetes mellitus. On physical examination, the patient was pale and had a holosystolic murmur. The patient's hemoglobin level was 5.5 g/dL. Capsule endoscopy (CE) localized the source of bleeding to multiple sites near the terminal ileum. Due to ongoing hematochezia, the patient required daily blood transfusions. Colonoscopy with terminal ileal intubation and endoscopic therapy were attempted. However, these treatment strategies failed due to tortuosity and looping of the colon. Embolization was not performed as extravasation was not identified on abdominal CT angiography (CTA). Due to persistent bleeding, the patient underwent laparoscopic exploration and transillumination using a two-scope technique to identify angiodysplastic lesions. Given the extent of angiodysplasia, the laparoscopic exploration was converted to open surgery,100 cm of distal small bowel was resected, and a primary anastomosis was performed ( Fig. 2 ). The patient's postoperative course was complicated by ongoing gastrointestinal bleeding and the patient was taken to the operating room for intraoperative endoscopy, resection of an additional 35 cm of small bowel, and creation of an ileostomy. A sterile endoscope was passed into a blood-filled small bowel until a segment of bloodless small bowel was reached. The endoscope was then withdrawn until a bleeding angiodysplasia lesion was identified. Persistent bleeding at the ileostomy site required submucosal injection of epinephrine, APC (30 W and flow rate of 0.8 L/min), and the placement of an OTSC to control an actively bleeding Dieulafoy lesion. The patient was discharged from the hospital after 35 days. At the 2-year follow-up, gastrointestinal bleeding was not observed, and her anemia had resolved.

## Analysis of Cases Reported in the Literature

Our computer search between 2000 and 2020 identified five investigators who published their experience with intraoperative endoscopy to enhance the success of surgical resection ( Table 1 ). In total, 21 patients were reported to undergo intraoperative endoscopic-guided bowel resection for angiodysplasia. Both laparoscopic and open surgical resections complemented by intraoperative endoscopy have been reported. Moreover, 7 of 21 published studies included the demographic characteristics of the reported patients. The average age of those seven patients was 74 years. Among the seven patients, four were men. The angiodysplasia lesions were located in the jejunum in 18 of 21 patients and in the colon in 3 of 21 patients. The length of the resected bowel ranged from 10 to 200 cm in the reported literature. Valleur's group 6 has been specifically interested in intraoperative endoscopy as an approach to decrease rebleeding rates following surgical resection; they report 25 patients with gastrointestinal bleeding who underwent endoscopic-guided bowel resection. Among them, 14 patients were diagnosed with angiodysplasia of the small intestine ( *n* = 11) and colon ( *n* = 3). 6 Only 1 of these 14 patients presented with postoperative bleeding. In the literature, only 2 of 21 patients returned with a rebleeding episode.

## Discussion

GIADs are rare, arborized AVMs that frequently result in gastrointestinal bleeding. Occasionally GIADs are associated with massive hemorrhage resistant to multimodal medical treatment, as in the above-mentioned cases. Previously, these conditions were believed to be most commonly located within the right ascending colon. However, they can be found throughout the entire gastrointestinal tract. 1 Jackson et al reported that approximately 10% of patients with a history of obscure gastrointestinal bleeding and greater than one AVM endoscopically had additional GIADs on CE 1 . Further, greater than 50% of these lesions were located in the small bowel, with 37% located in the jejunum and 15% located in the ileum. Bollinger et al specifically reported that the majority of GIADs are found in the proximal small bowel, as was true of the patient in our first case. 8 The diagnostic approaches to identify GIADs include CE, deep endoscopy, colonoscopy, abdominal CTA, and red blood cell scintigraphy. 9 However, based on the initial studies deployed, missed lesions may be a cause of recurrent disease. As an example, in symptomatic patients with an initially negative CE, 29% of patients re-presented and were found to have GIADs on repeat CE. 1 Thus, a comprehensive diagnostic workup is important to prevent recurrent bleeding secondary to missed, untreated lesions. Specifically, Jackson et al described success in the use of Endocuff-assisted push enteroscopy to reach distal angiodysplastic lesions and prevent missed GIAD lesions. 10 In most patients, bleeding from GIADs resolves spontaneously after diagnosis. However, comorbidities including aortic stenosis, chronic kidney disease, coagulopathic conditions, or chronic anticoagulation can all increase the risk of recurrent bleeding and the development of new GIADs. 11

Medical therapy, endoscopic treatment, catheter-based embolization, or surgical resection of the involved bowel is frequently considered in patients whose condition does not resolve spontaneously. Endoscopic treatment using APC, clip placement, and electrocoagulation is an initially effective management strategy in most patients. Speaking of this, Gutierrez et al found that recurrent GIADs can typically be managed with deep enteroscopy with an Endocuff in combination with iron repletion, rarely requiring eventual surgical management. 12 However, eventually up to 45% of patients, particularly those with GIADs in the small intestine, may experience rebleeding at 1 to 2 years following therapy. 11 Medical management with somatostatin analogs and antiangiogenics may be effective as part of the initial therapy. For example, octreotide has lower rates of rebleeding compared with placebo at the 1- and 2-year follow-ups. Nevertheless, 23% of patients on octreotide still presented with rebleeding GIADs within the first year, and 32% developed rebleeding within the first 2 years. 13 Ultimately, patients who fail medical and endoscopic management may require surgical resection. Intraoperative endoscopy can facilitate the visualization and treatment of additional dysplastic lesions during surgery, as in the case of our two patients.

Before initiating any treatment modality, the underlying comorbidities that may affect disease process, efficacy of medical therapy, and clinical decision-making should be considered. For example, there is a robust body of literature that suggests a possible association between angiodysplasia and aortic stenosis, which was a preexisting condition in our second patient. Lourdusamy et al revealed that the prevalence rate of aortic stenosis in patients with bleeding angiodysplasias ranges from 7 to 41%. 14 This relationship may be explained by Heyde's syndrome, a clinical association of aortic stenosis, GIAD, bleeding, and anemia as well as type 2A von Willebrand syndrome. 15 The immediate identification of this condition is important as transcatheter aortic valve replacement (TAVR), compared with the standard treatment for gastrointestinal bleeding, is essential in achieving long-term bleeding resolution. For example, Kubo et al recently reported a patient in whom blood transfusion resistant GIAD lesions in the presence of aortic stenosis raised concern for Heyde's syndrome. 16 Despite catheter embolization and bowel resection, the patient's bleeding persisted without resolution until TAVR was performed. Thus, the evaluation of concurrent aortic stenosis is warranted in patients with persistent gastrointestinal bleeding despite maximum treatment efforts. As medical treatment failure is common, there is a great interest in analyzing retrospective data and collecting case reports to elucidate better diagnostic and treatment strategies for GIADs.

The results of our analysis of the patients reported in the literature support the notion that occult bleeding most commonly occurs in the small intestine and that angiodysplasia of the colon can often be addressed by standard colonoscopy. Additionally, as only 2 of 21 patients had a rebleeding episode, intraoperative endoscopic-guided bowel resection appears to be a highly successful approach for patients with recalcitrant bleeding from angiodysplasia. Moreover, these findings are supported by our two cases, as bleeding resolved following endoscopic-guided surgical resection for both patients. This combined approach is well supported at our institution as we have a well-established multidisciplinary collaboration in the care of our patients with complex gastrointestinal conditions.

For the past 10 years at our institution, we have held a weekly 1-hour joint conference for members of the general surgery service and advanced endoscopy service to discuss optimal therapy, which often involves combined procedures. This conference has led to multiple intraoperative collaborations and solutions for complex patient issues, including bowel resection with intraoperative endoscopy as exemplified by the two patients described in our report. These two patients were originally discussed at this conference, and decisions were made at that time to conduct intraoperative endoscopic-guided bowel resections. Sterile colonoscopes were utilized, and the advanced endoscopy team scrubbed in donning surgical attire. The benefit of this collaboration was the identification of actively bleeding GIADs via intraoperative endoscopy. Using this technique, other sources of gastrointestinal bleeding may also be detected to prevent failure in controlling hemorrhage. For example, in the case of our second patient, a Dieulafoy lesion was identified and successfully controlled with intraoperative endoscopic therapy. The additional benefit of this combined approach is the excellent closed-loop care that can be provided throughout the preoperative, postoperative, and follow-up periods by members of the gastroenterology service who were directly involved in the surgical intervention. A laparoscopic approach at our institution, which can be used to identify small angiodysplasia lesions by transillumination, requires the use of two laparoscopes. The first laparoscope shines light through the bowel and the second laparoscope is used to image the transilluminated intestine.

Combined intraoperative endoscopy and bowel resection is effective and beneficial for the management of multimodal treatment-resistant GIADs. Nonetheless, this approach has several limitations. First, the number of studies utilizing this method is limited. Second, the availability of advanced endoscopy specialists who can perform intraoperative endoscopy at some institutions may be limited. Third, bowel resection may not be recommended for patients who are acutely ill or who cannot tolerate surgical procedures. Thus, further investigation should be conducted to explore alternative, nonsurgical treatment options for patients with treatment-resistant GIADs.

Although the mechanisms of GIAD formation are not completely understood, new treatments targeting these acquired vascular malformations are currently being evaluated. More specifically, therapies targeting the inhibition of angiogenesis have shown promise. For example, bevacizumab is a recombinant, humanized monoclonal antibody that selectively inhibits circulating vascular endothelial growth factor (VEGF), an angiogenic protein. Baldeosingh et al demonstrated that patients with GIAD who had a previous history of failed endoscopic therapy had decreased angiogenesis, improved hemoglobin levels, decreased transfusion requirements, and decreased hospitalizations following the administration of bevacizumab. 17 Similarly, Ge et al found that thalidomide, a VEGF inhibitor, was associated with decreased bleeding, blood transfusion requirements, and VEGF levels in patients with GIAD. 18 However, both bevacizumab and thalidomide are teratogens and are associated with significant side effects, which may limit their therapeutic use. 17 18 Some patient populations, such as those outside of childbearing ages and otherwise healthy patients, may benefit from these treatment options. Overall, promising therapies specifically targeting and inhibiting angiogenesis pathways are currently being evaluated, which could benefit patients with treatment-resistant GIADs who may not qualify for bowel resection.

## Conclusion

In rare patients with persistent gastrointestinal bleeding from angiodysplasia, in whom standard medical and endoscopic therapies fail, collaborative intraoperative endoscopy to guide surgical bowel resection can be an efficacious approach.
